# The clinical efficacy of type 2 monoclonal antibodies in eosinophil-associated chronic airway diseases: a meta-analysis

**DOI:** 10.3389/fimmu.2023.1089710

**Published:** 2023-04-11

**Authors:** Yuan Wu, Mengfen Huang, Jinyao Zhong, Yue Lu, Kao Gan, Rongyuan Yang, Yuntao Liu, Jiqiang Li, Jiankun Chen

**Affiliations:** ^1^ The Second Clinical Medical College, Guangzhou University of Chinese Medicine, Guangzhou, China; ^2^ Guangzhou University of Chinese Medicine, Guangzhou, China; ^3^ The Second Affiliated Hospital of Guangzhou University of Chinese Medicine (Guangdong Provincial Hospital of Chinese Medicine), Guangzhou, China; ^4^ Guangzhou Key Laboratory of Traditional Chinese Medicine for Prevention and Treatment of Emerging Infectious Diseases, Guangzhou, China

**Keywords:** eosinophil-associated chronic airway diseases, efficacy, randomized controlled trials, meta-analysis, type 2 monoclonal antibodies

## Abstract

**Background:**

Anti-type 2 inflammation therapy has been proposed as a treatment strategy for eosinophil-associated chronic airway disorders that could reduce exacerbations and improve lung function. We performed a meta-analysis of randomized controlled trials to assess the effectiveness of type 2 monoclonal antibodies (anti-T2s) for eosinophil-associated chronic airway disorders.

**Methods:**

PubMed, Embase, Web of Science, and Cochrane Library were searched from their inception to 21 August 2022. Randomized clinical trials evaluating the effectiveness of anti-T2s versus placebo in the treatment of chronic airway diseases were selected. The outcomes were exacerbation rate and change in pre-bronchodilator forced expiratory volume in 1 s (FEV1) from baseline. The Cochrane Risk of Bias Assessment Tool 1.0 was used to evaluate the risk of bias, and the random-effects or fixed-effect model were used to pool the data.

**Results:**

Thirty-eight articles concerning forty-one randomized clinical trials with 17,115 patients were included. Compared with placebo, anti-T2s therapy yielded a significant reduction in exacerbation rate in COPD and asthma (Rate Ratio (RR)=0.89, 95%CI, 0.83-0.95, I^2 =^ 29.4%; RR= 0.59, 95%CI, 0.52-0.68, I^2 =^ 83.9%, respectively) and improvement in FEV1 in asthma (Standard Mean Difference (SMD)=0.09, 95%CI, 0.08-0.11, I^2 =^ 42.6%). Anti-T2s therapy had no effect on FEV1 improvement in COPD (SMD=0.05, 95%CI, -0.01-0.10, I^2 =^ 69.8%).

**Conclusion:**

Despite inconsistent findings across trials, anti-T2s had a positive overall impact on patients’ exacerbation rate in asthma and COPD and FEV1 in asthma. Anti-T2s may be effective in treating chronic airway illnesses related to eosinophils.

**Systematic Review Registration:**

https://www.crd.york.ac.uk/PROSPERO/, identifier CRD42022362280.

## 1 Introduction

Chronic airway diseases pose a serious public health risk, causing 3.91 million deaths in 2017, accounting for 7% of all death worldwide, which is mainly attributable to chronic obstructive pulmonary disease (COPD) and asthma (1).

Elevated blood eosinophils, sputum eosinophils, or exhaled breath nitric oxide fraction (FeNO) are common manifestations of eosinophilic airway inflammation, which are associated with increased risk of patient complications, recurrent acute exacerbations, pneumonia, prolonged hospitalization, and increased morbidity and mortality (2–9). Patients with persistent eosinophilic airway inflammation may benefit from inhaled glucocorticosteroids (ICS) (10–14). Nevertheless, long-term ICS therapy may result in several unfavorable adverse events, such as osteoporosis, diabetes, cataracts, and higher infection risk (15, 16). Additionally, ICS is not always well-tolerated by patients.

Several monoclonal antibodies targeting particular inflammatory pathways have been created to address the complications mentioned above. Pathogenic factor-induced cellular release of cytokines, including interleukin (IL)-4, IL-5, IL-9, IL-13, IL-25, IL-33, immunoglobulin E (IgE) and thymic stromal lymphopoietin (TSLP) are closely related to eosinophilic airway inflammation (17, 18). Except for blocking the downstream targets, activation of toll-like receptor 9 (TLR9) has been shown to balance the T helper (Th) 1/Th2 axis (19). Type 2 monoclonal antibodies (anti-T2s) are effective in decreasing FeNO and eosinophil levels (20–24). However, results from previous research, which investigated the effectiveness of anti-T2s in reducing exacerbation rate and improving lung function, have been controversial. Therefore, we performed a meta-analysis of randomized controlled trials (RCTs) to examine the efficacy of anti-T2s for chronic eosinophilic airway diseases, investigating the possibility of endotype-guided strategy in the management of chronic airway disorders.

## 2 Methods

### 2.1 Protocol

The study protocol was registered at the International Prospective Register of Systematic Reviews (number CRD42022362280).

### 2.2 Data sources and search strategies

PubMed, Embase, Web of Science, and Cochrane Library were searched from their inception to 21 August 2022. We used the following search strategy to find all studies evaluating anti-T2s, including IL-5, IL-4, IL-9, IL-13, IL-25, IL-33, IgE, TSLP, and TLR9 for patients with eosinophil-associated COPD and asthma: (mepolizumab OR reslizumab OR benralizumab OR tralokinumab OR lebrikizumab OR dupilumab OR anti-interleukin OR MEDI-528 OR GSK679586 OR omalizumab OR tezepelumab OR AZD1419 OR CYT003 OR itepekimab OR XKH001) AND (asthma OR chronic obstructive diseases). The detailed search strategy is shown in **Supplementary Table 1**. Languages had no restrictions. The pertinent review articles and their citations were also checked.

### 2.3 Study selection

Endnote X9 software was adopted to manage the eligible studies during the literature screening and automatically remove duplicate documents. The following particular inclusion criteria were met (1): Participants: individuals (6 years of age or older) with asthma or COPD who met one or more criteria for eosinophilic inflammation at study enrolment or within the previous year. (2) Interventions: with anti-IL-5, anti-IL-4, anti-IL-13, anti-IL-9, anti-IL-25, anti-IL-33, anti-TSLP, anti-IgE or TLR9 agonist therapy at any dose. (3) Randomized placebo-controlled trials. (4) Reporting the following outcomes: exacerbation rate and change in pre-bronchodilator forced expiratory volume in 1 s (FEV1) from baseline.

Excluded criteria were as follows: (1) Studies did not involve eosinophilic endotype. (2) Interventions were not related to type 2 inflammation. (3) Studies did not assess the exacerbation rate or FEV1. (4) Not RCTs or literature types were reviews, letters, second analysis, or conferences.

The source data, together with the rate ratio (RR) or mean difference (MD), are given or can be computed from the data. All references were independently reviewed by two authors (YW and MH) following the selection criteria. Any disagreements were resolved through conversation or by a third author (JL).

### 2.4 Data extraction and quality assessment

The preferred reporting items for systematic reviews and meta-analyses (PRISMA) statement was followed (25) (see **Supplementary Table 5**). Two authors (YW and MH) independently retrieved data from eligible studies using Excel 2019 in a standardized data extraction form in a blinded manner based on the authors, publication year, research design, patient characteristics (age, gender, etc.), the type of anti-T2s used, the dosage, the length of the therapy, the definitions of the outcomes, the exacerbation rate, and the change in FEV1. A third author (JL) was consulted to settle disagreements. Furthermore, we evaluated the risk of bias using Cochrane Risk of Bias Assessment Tool 1.0, which included sufficient sequence generation, allocation concealment, blinding of participants and staff, inadequate outcome data, selective reporting, and additional bias (26). Two senior researchers (RY and YL) evaluated the reliability of the evidence using the GRADE-profiler software (V.3.6, The GRADE Working Group, 2010), items including the risk of bias, inconsistency, indirectness, imprecision, and publication bias, and the evidence was assessed as 4 levels: high quality, moderate quality, low quality, and very low quality.

### 2.5 Statistical analysis

We conducted a series of meta-analysis to compare the efficacy of anti-T2s with a placebo. For dichotomous data (exacerbation rate), intervention effects were reported using RR and 95% confidence intervals (CI), whereas standard mean differences (SMD) and 95%CI were used for continuous data (FEV1). Following the Cochrane Handbook, we aggregated two or three intervention groups into a single intervention group when research demonstrates more than two arms (26). The chi-squared test and the I^2^ statistic were used to measure heterogeneity. Significant heterogeneity is indicated by an I^2^ value of more than 50% (27). When there was significant heterogeneity, a random effect model was applied, and meta-regression analyses were performed to investigate the possible origins of heterogeneity. Planned considerations included ages, risk of bias, and demographics (exacerbation history, type 2 inflammatory criteria, and so forth). In comparisons involving at least 10 trials, publication bias was examined using a funnel plot and Eggers’ test (28, 29). The influence of publication bias was estimated using the trim-and-fill method (30). Sensitivity analyses were carried out to assess the robustness of the overall effect sizes by removing one study at a time. Review Manager (V.5.4.1, The Cochrane Collaboration, 2020) and Stata (V.15.1) were used for all statistical analyses. A two-sided P value of 0.05 was considered statistically significant.

## 3 Results

### 3.1 Search results

**Figure 1:**
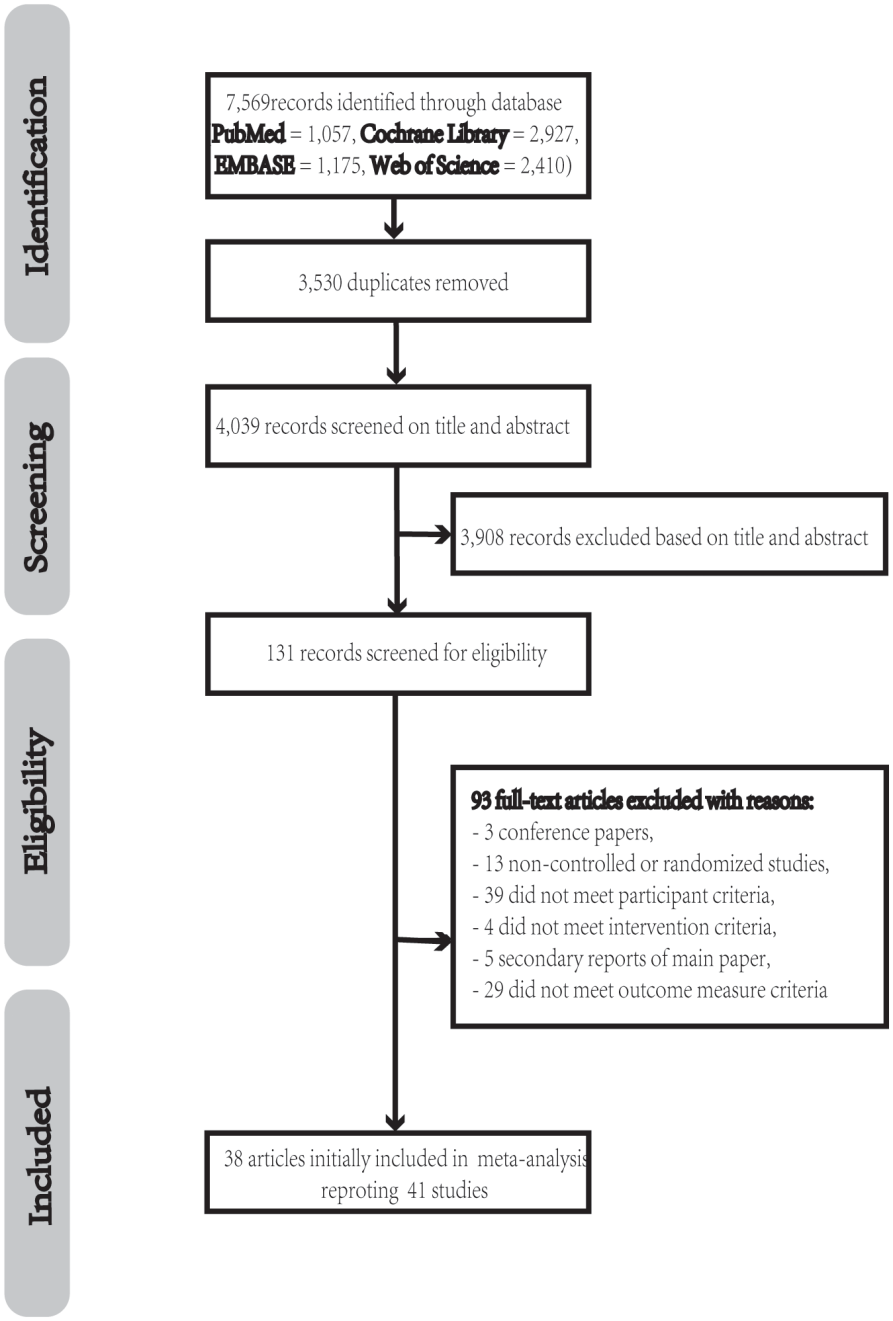
Flow chart of study identification, inclusion, and exclusion.

A total of 7569 potentially pertinent articles were found. 3530 duplicate records from among all the potential studies were eliminated, leaving 4039 papers for screening. We found and obtained 131 papers in full text for review after examining the titles and abstracts. Ninety-three of these publications were excluded due to the following reasons: improper population (n = 39) (31–69), incorrect intervention (n = 4) (70–73), improper outcomes (n =29) (74–102), non-RCT (n = 13) (103–115), conferences (n = 3) (116–118), and second analysis (n = 5) (119–123). In the end, this meta-analysis included 38 articles with 41 studies (**Figure 1**).

### 3.2 Description of included trials

Thirty-eight articles, covering forty-one trials with 17,115 individuals, were included (**Supplementary Table 2**). The number of subjects in the studies ranges from 61 to 1545. Eleven of these studies employed benralizumab (124–133), three reslizumab (23, 134, 135), two dupilumab (21, 136), one lebrikizumab (137), eight mepolizumab (22, 138–143), seven omalizumab (111, 144–149), two tezepelumab (150, 151), one astegolimab (152), one itepekimab (153), one AZD1419 (154), one quilizumab (155), one CYT003 (156) and two tralokinumab (157). The duration of the treatment ranges from 12 to 56 weeks, and the follow-up was 12 to 84 weeks. Six trials administered the monoclonal antibody by intravenous infusion (IV), thirty-three studies by subcutaneous (SC), one by inhalation, and one study comprised both IV and SC arms. Thirty-four studies included patients with asthma, whereas seven researches included those with COPD.

All included patients with COPD who had an exacerbation history. In thirty-four studies with asthma patients, seven studies included severe asthma, five studies included moderate to severe asthma, one study included mild to moderate asthma, twelve studies included refractory, uncontrolled, or persistent asthma, and the remaining studies did not specify asthma severity; patients in eight studies required medium to high dose ICS plus long-acting β2-agonists (LABA), two studies required at least medium ICS, one study required 6-month maintenance treatment with systemic glucocorticoids, and one study required not receiving ICS; seventeen studies required exacerbation history.

The definition of ‘type 2 inflammation’ varied among studies. Four studies were defined by FeNO levels, three studies were defined by a sputum eosinophil count, twenty-two studies were defined by baseline blood eosinophil counts, one study was defined by eosinophil counts in blood or sputum, one study was defined by baseline blood eosinophil counts or IgE levels, five by IgE levels, and one study defines by combinatorial biomarkers, including FeNO levels, eosinophil counts in blood or sputum, whereas four studies did not specify the criteria.

### 3.3 Efficacy outcomes

In chronic airway illnesses associated with eosinophils, we contrasted anti-T2s with a placebo. The primary outcome was the exacerbation rate. A COPD or asthma exacerbation was defined as a clinical worsening for at least three days, a temporary increase in the ICS background dosage, the need for systemic corticosteroid treatment, the consumption of antibiotics, hospitalization, or mortality resulting from an airway disease. The secondary endpoint was the change in FEV1 from baseline measured by spirometry. Since the number of studies on asthma is much higher than studies on COPD, to eliminate the influence, the population was divided into the asthma group and COPD group for meta-analysis, respectively.

#### 3.3.1 Exacerbation rate in COPD

**Figure 2:**
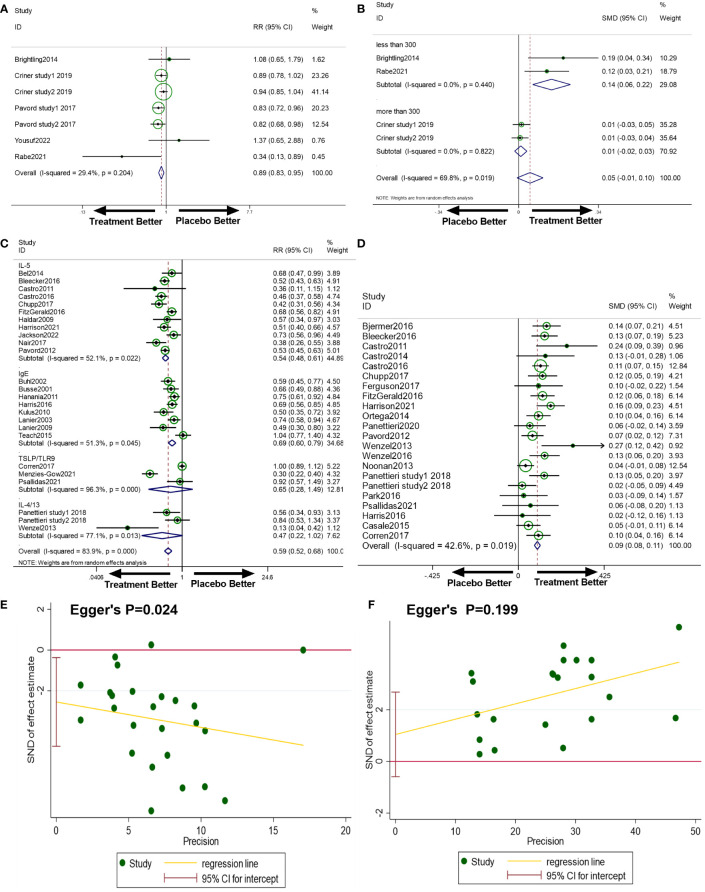
**(A)** The effect of anti-T2s versus placebo on exacerbation rate in COPD. Fixed-effect model. **(B)** The effect of anti-T2s versus placebo on FEV1 change from baseline in COPD. Random-effect model. **(C)** The effect of anti-T2s versus placebo on exacerbation rate in asthma. Random-effect model. **(D)** The effect of anti-T2s versus placebo on FEV1 change from baseline in asthma. Fixed-effect model. **(E)** Egger’s test of exacerbation rate in asthma in the meta-analysis. **(F)** Egger’s test of FEV1 in asthma in the meta-analysis. *CI*, confidence interval; SMD, standard mean difference; RR, rate ratio.

There were seven studies included to analyse anti-T2s’ efficacy in reducing the exacerbation rate of COPD. As a result, anti-T2s considerably reduced the exacerbation rate in COPD when compared to placebo (RR=0.89, 95%CI, 0.83-0.95, I^2 =^ 29.4%, **Figure 2A**).

#### 3.3.2 FEV1 in COPD

There were four studies included to analyse anti-T2s’ efficacy in improving FEV1 in COPD. As a result, anti-T2s improved pre-bronchodilator FEV1 in patients with COPD, whereas the difference was not statistically significant (SMD=0.05, 95%CI, -0.01-0.10, I^2 =^ 69.8%, **Figure 2B**). Considering the between-study heterogeneity, a subgroup analysis based on the sample size of studies was applied. In the subgroup analysis, studies with a sample size of less than 300 subjects exhibited anti-T2s’ efficacy in improving FEV1 (SMD=0.14, 95%CI, 0.06-0.22, I^2 =^ 0%), while studies with sample size of more than 300 patients showed no effect on FEV1 improvement (SMD=0.05, 95%CI, -0.01-0.10, I^2 =^ 0%).

#### 3.3.3 Exacerbation rate in asthma

There were twenty-five studies included. Anti-T2s considerably reduced the exacerbation rate in asthma when compared to placebo (RR=0.59, 95%CI, 0.52-0.68, I^2 =^ 83.9%, **Figure 2C**). Publication bias on Egger’s test was present in this analysis (P=0.024, **Figure 2E**). But further investigation using the trim-and-fill test showed that this publishing bias did not affect the estimations (ie, no trimming was done because the data was unchanged).

Anti-IL-5 treatment was associated with a decreased incidence of asthma exacerbation compared to placebo in the subgroup analysis for different targets (RR=0.54, 95%CI, 0.48-0.61, I^2 =^ 52.1%). Similarly, anti-IgE therapy achieved a reduction in exacerbation of asthma (RR=0.69, 95%CI, 0.60-0.79, I^2 =^ 51.3%). The exacerbation rate was found decreasing with anti-IL-4/13, anti-TSLP, or TLR9 agonist medication when compared to placebo, although the difference was not statistically significant (RR=0.47, 95%CI, 0.22-1.02, I^2 =^ 77.1%; RR=0.65, 95%CI, 0.28-1.49, I^2 =^ 96.3%). Since the heterogeneity was partially decreased in subgroup analysis, different targets did not completely account for the between-study heterogeneity.

Univariable meta-regression using a random-effects model was performed and the results revealed that the criteria of ‘type 2 inflammation’, history of exacerbation, age, sample size, risk bias, severity, atopy, and different targets were not significantly associated with heterogeneity related to the exacerbation rate in asthma (**Supplementary Table 3**).

#### 3.3.4 FEV1 in asthma

Data on pre-bronchodilator FEV1 were reported from twenty-two trials, of which seventeen reported a change in FEV1 from baseline, four reported a change in FEV1% from baseline, and one reported both.

Anti-T2s was associated with a substantial improvement in FEV1 change from baseline in a pooled analysis of twenty-two trials (SMD=0.09, 95%CI, 0.08-0.11, P<0.001, **Figure 2D**) with acceptable heterogeneity (I^2 =^ 42.6%, P=0.019). No publication bias existed (Egger’s P=0.199, **Figure 2F**).

### 3.4 Risk of bias

**Figure 3:**
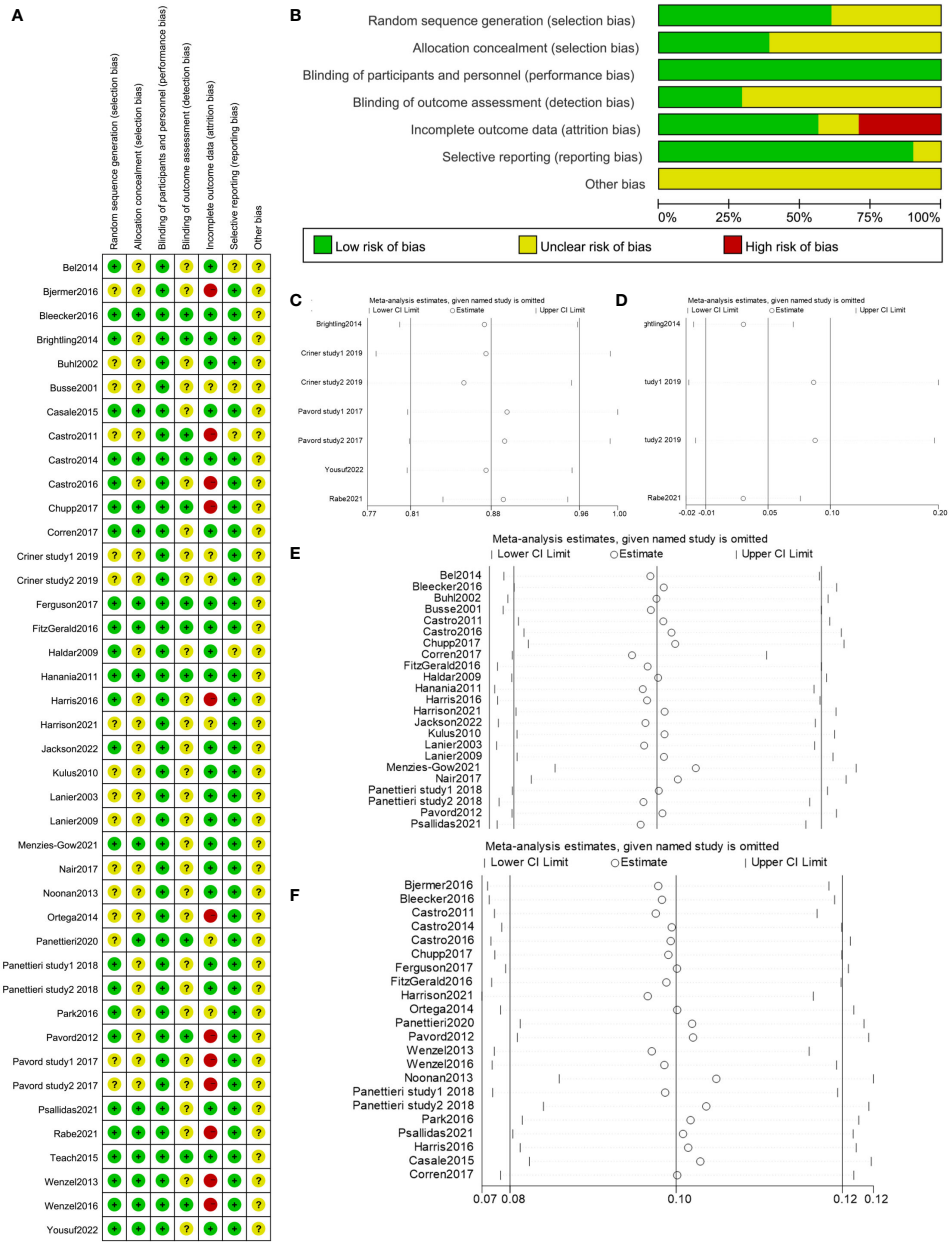
**(A, B)** Risk of bias summary of included studies. **(C)** Sensitivity analysis of exacerbation rate in COPD in the meta-analysis. **(D)** Sensitivity analysis of change in FEV1 from baseline in COPD in the meta-analysis. **(E)** Sensitivity analysis of exacerbation rate in asthma in the meta-analysis. **(F)** Sensitivity analysis of change in FEV1 from baseline in asthma in the meta-analysis.

A total of 25 researches (61.0%) adequately explained the randomization process. In 16 researches (39.0%), the random allocation was acknowledged, while in 25 studies (61.0%), allocation concealment was unclear. Research blinding was used in all of the investigations. 11 studies (26.8%) were at low risk for the outcome assessment’s blinding. There was a low risk to the integrity of the outcome data in 23 trials (56.1%). There was a low risk of selection bias for 37 (90.2%). The data from each study was insufficient to determine if the risk of other biases was low or high (**Figures 3A, B**). In 32 studies included to assess the exacerbation rate, 20 studies in total (62.5%) provided a comprehensive explanation of the randomization procedure. In 11 investigations (34.4%), the random allocation was confirmed, while allocation concealment was uncertain in 21 studies (65.6%). In each study, research blinding was performed. 7 studies (21.9%) had a low risk of blinding in outcome assessment. In 19 trials (59.4%), there was a low risk in the integrity of the outcome data. For 28 studies (87.5%), the risk of selection bias was low. In 26 studies included to assess FEV1, 18 studies (69.2%) in total provided a thorough explanation of the randomization procedure. In 12 investigations (46.2%), the random allocation was acknowledged, and allocation concealment was ambiguous in 14 studies (53.8%). In each study, research blinding was applied. 9 studies (34.6%) had a low risk of blinding outcome assessment. In 11 trials (42.3%), there was a low risk of the integrity of the outcome data. 25 studies (96.2%) had a low risk of selective bias.

### 3.5 Sensitivity analysis

By removing one study at a time, sensitivity analyses were utilized to examine the impact of each study on the combined results. The outcome demonstrated that there had been no appreciable changes to the results’ stability (**Figures 3C–F**), which supported the validity and dependability of our analysis.

### 3.6 Certainty of the evidence

Because of the considerable heterogeneity and inconsistent findings across trials, the evidence received a low-quality level in exacerbation rate in asthma and change in FEV1 from baseline in COPD, and a moderate-quality level in exacerbation rate in COPD and change in FEV1 from baseline in asthma (**Supplementary table 4**).

## 4 Discussion

This meta-analysis included 41 RCTs from 38 articles with 17,115 participants and investigated the effect of anti-T2s in patients with chronic eosinophilic airway diseases on exacerbation rate and FEV1. Our results showed that anti-T2s significantly reduces exacerbation rate in COPD and asthma, and improve FEV1 in asthma when compared to placebo, indicating that type 2 chronic airway disease patients can benefit from endotype-guided therapy as a treatment option.

Exacerbations are far more likely to cause morbidity and mortality (158, 159). One of the main objectives of chronic airway illness management is to reduce the exacerbation rate (22, 127). According to earlier investigations, increased blood and sputum eosinophilic counts are independent risk factors for exacerbations (160–162). Anti-T2s reduce the FeNO, eosinophil cationic protein, and eosinophil levels in airway inflammation (21–24), indicating that airway eosinophilia is a novel target, thus anti-T2s may be a potential approach to chronic eosinophilic airway disorders treatment. Our meta-analysis, which revealed a marked decline in the exacerbation rate in both COPD and asthma, validated the claim. Patients with COPD receiving anti-T2s medication in contrast to placebo experienced a reduced exacerbation. Although individuals with asthma receiving anti-T2s therapy had decreased exacerbations than those receiving a placebo, the heterogeneity was statistically significant. A subgroup analysis was conducted according to different targets, which revealed inconsistent results among subgroups. Anti-IL-5 and anti-IgE therapy both achieved a reduction in asthma exacerbation with acceptable heterogeneity, while anti-IL-4/13, anti-TSLP, and TLR9 agonist therapy had a decreased trend of exacerbations than placebo, and the difference was not statistically significant. The following factors may account for the inconsistent results among subgroups: (1) The inclusion criteria for each study within the current meta-analysis varied, which may have led to significant heterogeneity among study populations in terms of exacerbation risk, eosinophil count, and disease severity; (2) Different therapy regimens were varied; (3) Anti-IL-4/13 treatment has shown a less consistently positive impact on the exacerbation rate, as the previous study reported (163). Tralokinumab, an anti-IL-13 agent, did not affect the exacerbation rate in the study of Panettieri et al, but another cohort in the same article showed a statistically significant reduction in exacerbations (157). This may be an indication of the ambiguous impact of blocking the IL4/13 pathway on reducing the exacerbation rate in asthma (4). Anti-TSLP and TLR9 agonist therapy had fewer studies to evidence their efficacy, and the existing studies varied in ages of participants, disease severity, and so forth, resulting in obvious heterogeneity. However, the overall beneficial impact of anti-T2s in reducing asthma exacerbations is consistent across the meta-analysis, despite some lingering confounding factors.

The crucial identifying feature of chronic airway illnesses in the clinic and pathology is airflow limitation. The lung function test continues to be the gold standard in diagnosis and a crucial indicator of management, which is typically measured by the change in FEV1 (164). The results on FEV1 of asthma and COPD are inconsistent. Even though FEV1 in COPD patients improved, the difference was not statistically significant, which was consistent with previous studies (165–167). Overall, FEV1 considerably improved in asthma patients receiving anti-T2s. FEV1 alone may not be the optimum assessment for the management of chronic airway illnesses. The bias in results may be caused by variations in race, medication dosage, or even trial participants’ status and severity. The conflicting results between asthma and COPD could be attributed to the varying baseline FeNO, blood, or sputum eosinophilia thresholds. In addition, it was observed that former smokers achieved more pronounced benefits than current smokers in a prespecified subgroup analysis, which might be explained by a broad pro-inflammatory effect of cigarette smoke, indicating that smoking status had an impact on the effectiveness of anti-T2s in treating COPD (50). Further research should be done to determine the COPD-specific threshold of type 2 inflammation and explore the effect of anti-T2s in COPD patients with different smoking status to address these deficiencies.

According to the Cochrane handbook, ambiguous allocation concealment might exaggerate the estimated effect in subjective outcomes, while the bias in objective outcomes is not confirmed (26). In our study, the outcomes, including exacerbation rate and FEV1, are tending to be objective, and the impact of ambiguous allocation concealment remains unclear. Meanwhile, the GRADE system was applied to evaluate the reliability of our results, which had considered the bias judgments.

## 5 Limitations

Some potential restrictions must be taken into account. Firstly, it is difficult to determine the influence of the severity and initial therapy of included patients on the outcomes of the investigations. Secondly, a few of the research was conducted on a limited scale, which would limit their ability to investigate the true outcome. Thirdly, we failed to investigate the potential impact of disease severity, gender, and body mass index on outcomes given the limited data available. Fourthly, due to the finite number of specifically aimed at IL-4/13 pathway targeting, we were unable to further compare the effects of anti-IL-4 and anti-IL-4/13 treatment in subgroup analysis for exacerbation rate. Additionally, RCTs related to the anti-IL-9 agent were not included due to not meeting the inclusion criteria, RCTs related to anti-IL-25 therapy were in progress (NCT05128409), and RCTs of anti-T2s on COPD were under publication (NCT03615040, NCT03930732, NCT04456673). Finally, although using various intervention dosages and administration techniques, as recommended by the Cochrane handbook, we combined two or three intervention groups into a single arm, making it difficult to establish the ideal dosage. We should also be aware of the fact that different studies used various definitions of ‘type 2 inflammation’, and because no study included data on specific patients, we were unable to further examine the correlation between baseline levels of eosinophils or FeNO and treatment outcomes.

## 6 Conclusions

The current meta-analysis concluded that anti-T2s could considerably lessen exacerbations of chronic airway disorders. Therefore, anti-T2s may be effective in treating chronic airway illnesses associated with eosinophils. The findings highlight the effectiveness of endotype-guided treatment in chronic eosinophilic airway inflammation illnesses regardless of various background therapies and ‘type 2 inflammation’ criteria.

## Data availability statement

The original contributions presented in the study are included in the article/**Supplementary Material**. Further inquiries can be directed to the corresponding authors.

## Author contributions

YW, JL, and JC conceived this meta-analysis. YW, MH, and JZ extracted the data and wrote the manuscript. YLu did statistical analyses and checked them. KG helps the methods. RY and YLi evaluated the reliability of the evidence. YLu, RY, YLi, JL, and JC revised the manuscript. All authors contributed to the article and approved the submitted version.
