# Supplementary material for: The clinical efficacy of type 2 monoclonal antibodies in eosinophil-associated chronic airway diseases: a meta-analysis
Source: Front Immunol. 2023 Apr 11;14:1089710. doi: 10.3389/fimmu.2023.1089710 (PMC10126252; doi:10.3389/fimmu.2023.1089710)
Supplement: Supplementary file 1 [file DataSheet_1.docx]

Supplementary Table 1 Search strategies

| **PubMed** |
| --- |
| #1  COPD[Title/Abstract] OR COAD[Title/Abstract] OR COBD[Title/Abstract] OR chronic obstructive pulmonary disease[Title/Abstract] OR pulmonary disease, chronic obstructive[Title/Abstract] OR chronic obstructive pulmonary disorder[Title/Abstract] OR obstructive pulmonary disease[Title/Abstract] OR pulmonary disease, chronic obstructive[Title/Abstract] OR pulmonary disorder, chronic obstructive[Title/Abstract] OR chronic obstructive lung disease[Title/Abstract] OR chronic obstructive lung disorder[Title/Abstract] OR lung chronic obstructive disease[Title/Abstract] OR lung disease, chronic obstructive[Title/Abstract] OR lung diseases, obstructive[Title/Abstract] OR obstructive lung disease[Title/Abstract] OR obstructive lung disease, chronic[Title/Abstract] OR airflow obstruction, chronic[Title/Abstract] OR airflow obstructions, chronic[Title/Abstract] OR chronic airflow obstructions[Title/Abstract] OR chronic airflow obstruction[Title/Abstract] OR chronic obstructive airway disease[Title/Abstract] OR chronic obstructive bronchitis[Title/Abstract] OR chronic obstructive bronchopulmonary disease[Title/Abstract] OR chronic obstructive respiratory disease[Title/Abstract] OR obstructive respiratory disease[Title/Abstract] OR obstructive respiratory tract disease[Title/Abstract] OR pulmonary disease, chronic obstructive[Mesh]  #2  Asthma[Title/Abstract] OR Asthmas[Title/Abstract] OR Bronchial Asthma[Title/Abstract] OR Asthma, Bronchial OR Asthma[Mesh]  #3  epolizumab[Title/Abstract] OR bosatria[Title/Abstract] OR nucala[Title/Abstract] OR sb 240563[Title/Abstract] OR sb-240563[Title/Abstract] OR sb240563[Title/Abstract] OR Reslizumab[Title/Abstract] OR cinqaero[Title/Abstract] OR cinqair[Title/Abstract] OR sch 55700[Title/Abstract] OR sch55700[Title/Abstract] OR Benralizumab[Title/Abstract] OR fasenra[Title/Abstract] OR medi 563[Title/Abstract] OR medi563[Title/Abstract] OR Tralokinumab[Title/Abstract] OR CAT-354[Title/Abstract] OR Lebrikizumab[Title/Abstract] OR LY3650150[Title/Abstract] OR Dupilumab[Title/Abstract] OR GSK679586[Title/Abstract] OR MEDI-528[Title/Abstract] OR anti-interleukin[Title/Abstract] OR anti-IL[Title/Abstract] OR antibodies, monoclonal[Title/Abstract] OR antibody, monoclonal[Title/Abstract] OR monoclonal antibody[Title/Abstract] OR antibodies, monoclonal, humanized[Title/Abstract] OR antibodies, monoclonal, murine derived[Title/Abstract] OR antibodies, monoclonal, murine-derived[Title/Abstract] OR clonal antibody[Title/Abstract] OR hybridoma antibody[Title/Abstract] OR monoclonal antibodies[Title/Abstract] OR Antibodies, Monoclonal[Mesh] OR IgE [Title/Abstract] OR omalizumab [Title/Abstract] OR TSLP [Title/Abstract] OR tezepelumab [Title/Abstract] OR TLR9 [Title/Abstract] OR AZD1419 [Title/Abstract] OR CYT003 [Title/Abstract] OR IL33 [Title/Abstract] OR [Title/Abstract] OR itepekimab [Title/Abstract] OR IL25 [Title/Abstract] OR IL17E [Title/Abstract] OR XKH001 [Title/Abstract]  #4  (randomized controlled trial[Publication Type]) OR (randomized[Title/Abstract])) OR placebo[Title/Abstract])  #5  (#1 OR #2) AND #3 AND #4 |
| **EMBASE** |
| #1'chronic obstructive lung disease'/exp  #2'copd':ti,ab  #3'coad':ti,ab  #4'cobd':ti,ab  #5'chronic obstructive pulmonary disease':ti,ab  #6'pulmonary disease, chronic obstructive':ti,ab  #7'chronic obstructive pulmonary disorder':ti,ab  #8'pulmobstructive pulmonary disease':ti,ab  #9'pulmonary disease, chronic obstructive':ti,ab  #10'pulmonary disorder, chronic obstructive':ti,ab  #11'chronic obstructive lung disease':ti,ab  #12'chronic obstructive lung disorder':ti,ab  #13'lung chronic obstructive disease':ti,ab  #14'lung disease, chronic obstructive':ti,ab  #15'lung diseases, obstructive':ti,ab  #16'obstructive lung disease':ti,ab  #17'obstructive lung disease, chronic':ti,ab  #18'airflow obstruction, chronic':ti,ab  #19'airflow obstructions, chronic':ti,ab  #20'chronic airflow obstructions':ti,ab  #21'chronic airflow obstruction':ti,ab  #22'chronic obstructive airway disease':ti,ab  #23'chronic obstructive bronchitis':ti,ab  #24'chronic obstructive bronchopulmonary disease':ti,ab  #25'chronic obstructive respiratory disease':ti,ab  #26'obstructive respiratory disease':ti,ab  #27'obstructive respiratory tract disease':ti,ab  #28 #1 OR #2 OR #3 OR #4 OR #5 OR #6 OR #7 OR #8 OR #9 OR #10 OR #11 OR #12 OR #13 OR #14 OR #15 OR #16 OR #17 OR #18 OR #19 OR #20 OR #21 OR #22 OR #23 OR #24 OR #25 OR #26 OR #27  #29'asthma'/exp  #30'asthma':ti,ab  #31'asthmas':ti,ab  #32'bronchial asthma':ti,ab  #33'asthma, bronchial':ti,ab  #34 #29 OR #30 OR #31 OR #32 OR #33  #35'monoclonal antibody'/exp  #36'antibodies, monoclonal':ti,ab  #37'antibody, monoclonal':ti,ab  #38'monoclonal antibody':ti,ab  #39'antibodies, monoclonal, humanized':ti,ab  #40'antibodies, monoclonal, murine derived':ti,ab  #41'antibodies, monoclonal, murine-derived':ti,ab  #42'clonal antibody':ti,ab  #43'hybridoma antibody':ti,ab  #44'monoclonal antibodies':ti,ab  #45'anti-interleukin':ti,ab  #46'anti-il':ti,ab  #47'medi-528':ti,ab  #48'gsk679586':ti,ab  #49'dupilumab':ti,ab  #50'lebrikizumab':ti,ab  #51'ly3650150':ti,ab  #52'tralokinumab':ti,ab  #53'cat-354':ti,ab  #54'benralizumab':ti,ab  #55'fasenra':ti,ab  #56'medi 563':ti,ab  #57'medi563':ti,ab  #58'reslizumab':ti,ab  #59'cinqaero':ti,ab  #60'cinqair':ti,ab  #61'sch 55700':ti,ab  #62'sch55700':ti,ab  #63'mepolizumab':ti,ab  #64'bosatria':ti,ab  #65'nucala':ti,ab  #66'sb 240563':ti,ab  #67'sb-240563':ti,ab  #68'sb240563':ti,ab  #69'IgE':ti,ab  #70'omalizumab':ti,ab  #71'TSLP':ti,ab  #72'tezepelumab':ti,ab  #73'TLR9':ti,ab  #74'AZD1419':ti,ab  #75'CYT003':ti,ab  #76'IL33':ti,ab  #77'itepekimab':ti,ab  #78'IL25':ti,ab  #79'IL17E':ti,ab  #80'XKH001':ti,ab  #81 #35 OR #36 OR #37 OR #38 OR #39 OR #40 OR #41 OR #42 OR #43 OR #44 OR #45 OR #46 OR #47 OR #48 OR #49 OR #50 OR #51 OR #52 OR #53 OR #54 OR #55 OR #56 OR #57 OR #58 OR #59 OR #60 OR #61 OR #62 OR #63 OR #64 OR #65 OR #66 OR #67 OR #68 OR #69 OR #70 OR #71 OR #72 OR #73 OR #74 OR #75 OR #76 OR #77 OR #78 OR #79 OR #80  #82'randomized controlled trial'/exp  #83 #28 OR #34  #84 #81 AND #82 AND #83 |
| **Cochrane Library** |
| #1 MeSH descriptor: [Pulmonary Disease, Chronic Obstructive] explode all trees  #2 MeSH descriptor: [Asthma] explode all trees  #3 (COPD):ti,ab,kw  #4 (COBD):ti,ab,kw  #5 (COAD):ti,ab,kw  #6 (chronic obstructive pulmonary disease):ti,ab,kw  #7 (pulmonary disease, chronic obstructive):ti,ab,kw  #8 (chronic obstructive pulmonary disorder):ti,ab,kw  #9 (obstructive pulmonary disease):ti,ab,kw  #10 (pulmonary disease, chronic obstructive):ti,ab,kw  #11 (pulmonary disorder, chronic obstructive):ti,ab,kw  #12 (chronic obstructive lung disease):ti,ab,kw  #13 (chronic obstructive lung disorder):ti,ab,kw  #14 (lung chronic obstructive disease):ti,ab,kw  #15 (lung disease, chronic obstructive):ti,ab,kw  #16 (lung diseases, obstructive):ti,ab,kw  #17 (obstructive lung disease):ti,ab,kw  #18 (obstructive lung disease, chronic):ti,ab,kw  #19 (airflow obstruction, chronic):ti,ab,kw  #20 (airflow obstructions, chronic):ti,ab,kw  #21 (chronic airflow obstructions):ti,ab,kw  #22 (chronic airflow obstruction):ti,ab,kw  #23 (chronic obstructive airway disease):ti,ab,kw  #24 (chronic obstructive bronchitis):ti,ab,kw  #25 (chronic obstructive respiratory disease):ti,ab,kw  #26 (obstructive respiratory disease):ti,ab,kw  #27 (obstructive respiratory tract disease):ti,ab,kw  #28 (Bronchial Asthma):ti,ab,kw  #29 (Asthma, Bronchial):ti,ab,kw  #30 (Asthma):ti,ab,kw  #31 (chronic obstructive bronchopulmonary disease):ti,ab,kw  #32 (Asthmas):ti,ab,kw  #33 #1 OR #2 OR #3 OR #4 OR #5 OR #6 OR #7 OR #8 OR #9 OR #10 OR #11 OR #12  OR #13 OR #14 OR #15 OR #16 OR #17 OR #18 OR #19 OR #20 OR #21 OR #22 OR  #23 OR #24 OR #25 OR #26 OR #27 #29 OR #30 OR #31 OR #32  #34 (Mepolizumab):ti,ab,kw  #35 MeSH descriptor: [Antibodies, Monoclonal] explode all trees  #36 (bosatria):ti,ab,kw  #37 (nucala):ti,ab,kw  #38 (sb 240563):ti,ab,kw  #39 (sb-240563):ti,ab,kw  #40 (sb240563):ti,ab,kw  #41 (Reslizumab):ti,ab,kw  #42 (cinqaero):ti,ab,kw  #43 (cinqair):ti,ab,kw  #44 (sch 55700):ti,ab,kw  #45 (sch55700):ti,ab,kw  #46 (Benralizumab):ti,ab,kw  #47 (fasenra):ti,ab,kw  #48 (medi 563):ti,ab,kw  #49 (CAT-354):ti,ab,kw  #50 (Lebrikizumab):ti,ab,kw  #51 (LY3650150):ti,ab,kw  #52 (dupilumab):ti,ab,kw  #53 (GSK679586):ti,ab,kw  #54 (MEDI-528):ti,ab,kw  #55 (anti-interleukin):ti,ab,kw  #56 (anti-IL):ti,ab,kw  #57 (antibodies, monoclonal):ti,ab,kw  #58 (antibody, monoclonal):ti,ab,kw  #59 (monoclonal antibody):ti,ab,kw  #60 (antibodies, monoclonal, humanized):ti,ab,kw  #61 (antibodies, monoclonal, murine derived):ti,ab,kw  #62 (antibodies, monoclonal, murine-derived):ti,ab,kw  #63 (clonal antibody):ti,ab,kw  #64 (hybridoma antibody):ti,ab,kw  #65 (monoclonal antibodies):ti,ab,kw  #66 (Tralokinumab):ti,ab,kw  #67 (medi563):ti,ab,kw  #68 (IgE):ti,ab,kw  #69 (omalizumab):ti,ab,kw  #70 (TSLP):ti,ab,kw  #71 (tezepelumab):ti,ab,kw  #72 (TLR9):ti,ab,kw  #73 (AZD1419):ti,ab,kw  #74 (CYT003):ti,ab,kw  #75 (IL33):ti,ab,kw  #76 (itepekimab):ti,ab,kw  #77 (IL25):ti,ab,kw  #78 (IL17E):ti,ab,kw  #79 (XKH001):ti,ab,kw  #80 #34 OR #35 OR #36 OR #37 OR #38 OR #39 OR #40 OR #41 OR #42 OR #43 OR #44  OR #45 OR #46 OR #47 OR #48 OR #49 OR #50 OR #51 OR #52 OR #53 OR #54 OR  #55 OR #56 OR #57 OR #58 OR #59 OR #60 OR #61 OR #62 OR #63 OR #64 OR  #65 OR #66 OR #67 OR #68 OR #69 OR #70 OR #71 OR #72 OR #73 OR #74 OR #75 OR #76 OR #77 OR #78 OR #79  #81 #33 AND #80 |
| **Web of Science** |
| (TS=(COPD) OR TS=(COAD) OR TS=(COBD) OR TS=(chronic obstructive pulmonary disease) OR TS=(pulmonary disease, chronic obstructive) OR TS=(chronic obstructive pulmonary disorder) OR TS=(obstructive pulmonary disease) OR TS=(pulmonary disease, chronic obstructive) OR TS=(pulmonary disorder, chronic obstructive) OR TS=(chronic obstructive lung disease) OR TS=(chronic obstructive lung disorder) OR TS=(lung chronic obstructive disease) OR TS=(lung disease, chronic obstructive) OR TS=(lung diseases, obstructive) OR TS=(obstructive lung disease) OR TS=(obstructive lung disease, chronic) OR TS=(airflow obstruction, chronic) OR TS=(airflow obstructions, chronic) OR TS=(chronic airflow obstructions) OR TS=(chronic airflow obstruction) OR TS=(chronic obstructive airway disease) OR TS=(chronic obstructive bronchitis) OR TS=(chronic obstructive bronchopulmonary disease) OR TS=(chronic obstructive respiratory disease) OR TS=(obstructive respiratory disease) OR TS=(obstructive respiratory tract disease) OR TS=(Asthma) OR TS=(Asthmas) OR TS=(Bronchial Asthma) OR TS=(Asthma, Bronchial)) AND (TS=(Mepolizumab) OR TS=(bosatria) OR TS=(nucala) OR TS=(sb 240563) OR TS=(sb-240563) OR TS=(sb240563) OR TS=(Reslizumab) OR TS=(cinqaero) OR TS=(cinqair) OR TS=(sch 55700) OR TS=(sch55700) OR TS=(Benralizumab) OR TS=(fasenra) OR TS=(medi 563) OR TS=(medi563) OR TS=(Tralokinumab) OR TS=(CAT-354) OR TS=(Lebrikizumab) OR TS=(LY3650150) OR TS=(dupilumab) OR TS=(GSK679586) OR TS=(MEDI-528) OR TS=(anti-interleukin) OR TS=(anti-IL) OR TS=(antibodies, monoclonal) OR TS=(antibody, monoclonal) OR TS=(monoclonal antibody) OR TS=(antibodies, monoclonal, humanized) OR TS=(antibodies, monoclonal, murine derived) OR TS=(antibodies, monoclonal, murine-derived) OR TS=(clonal antibody) OR TS=(hybridoma antibody) OR TS=(monoclonal antibodies) OR TS=(IgE) OR TS=(omalizumab) OR TS=(TSLP) OR TS=(tezepelumab) OR TS=(TLR9) OR TS=( AZD1419) OR TS=(CYT003) OR TS=(IL33) OR TS=(itepekimab) OR TS=(IL25) OR TS=(IL17E) OR TS=(XKH001)) AND (TS=(randomized controlled trial) OR TS=(randomized) OR TS=(placebo) ) |

Supplementary Table 2: Characteristics of included studies

| Study | No. of subjects | Drug | Dose | Routine | Study population | Criteria to assess 'Type 2 inflammation' | Exacerbation definition ^a^ | Treatment duration, wks | Follow-up, wks | Age, years | Gender, female/male |
| --- | --- | --- | --- | --- | --- | --- | --- | --- | --- | --- | --- |
| Bel2014 | 135 | mepolizumab | 100mg q4w | SC | asthma; had at least a 6-month history of maintenance treatment with systemic glucocorticoids; eosinophilic inflammation; | baseline blood eosinophil counts≥0.300 cells/mL during the 12 months before screening or ≥0.150 cells/ml during the optimization phase | ① | 20 | 32 | ≥12 | 74/61 |
| Bjermer2016 | 315 | reslizumab | 0.3mg/kg q4w/ 3.0mg/kg q4w | IV | inadequately controlled asthma; treated with at least a medium-dose ICS; eosinophilic inflammation; | baseline blood eosinophil counts≥0.400 cells/mL | ① or ② | 16 | 20 | 12-75 | 174/131 |
| Bleecker2016^b^ | 809 | benralizumab | 30mg q4w/q8w | SC | asthma; at least two exacerbations while on high-dosage ICS and LABA in the previous year; eosinophilic inflammation; | baseline blood eosinophil counts≥0.300 cells/mL | ① or ④ | 48 | 48 | 12-75 | 527/282 |
| Brightling2014 | 101 | benralizumab | 100mg q8w | SC | moderate-to-severe COPD; at least one acute exacerbation requiring oral corticosteroids, antibiotics, or hospital admission in the past year; eosinophilic inflammation; | a sputum eosinophil count of 3.0% or more | ⑨ | 48 | 80 | 40-85 | 37/64 |
| Castro2011 | 106 | reslizumab | 3.0mg/kg q4w | IV | asthma; treated with high-dose ICS combination with at least one other agent; poorly controlled; eosinophilic inflammation; | a sputum eosinophil count of 3.0% or more | ① or ② | 12 | 15 | 18-75 | 63/43 |
| Castro2014^b^ | 324 | benralizumab | 2mg/20mg/100mg q8w | SC | uncontrolled asthma; two to six exacerbations in the past year; eosinophilic inflammation; | FeNO ≥ 50 ppb | NR | 52 | 52 | 18-75 | 219/105 |
| Castro2016 | 953 | reslizumab | 3.0mg/kg q4w | IV | inadequately controlled asthma; treated with at least a medium-dose ICS; eosinophilic inflammation; | baseline blood eosinophil counts≥0.400 cells/mL | ① or ③ | 52 | 65 | 12-75 | 597/356 |
| Chupp2017 | 551 | mepolizumab | 100mg q4w | SC | severe eosinophilic asthma; at least two exacerbations requiring treatment in the previous 12 months; | baseline blood eosinophil counts≥0.150 cells/mL at screening or ≥0.300 cells/ml at some time during the previous year | ① | 20 | 24 | ≥12 | 325/226 |
| Criner study1 2019^b^ | 1120 | benralizumab | 30mg/100mg q8w | SC | COPD; had frequent exacerbations despite receiving guideline-based inhaled treatment; eosinophilic inflammation; | baseline blood eosinophil counts≥220 per cubic millimeter | ⑧ | 56 | 56 | 40-85 | 328/792 |
| Criner study2 2019^b^ | 1545 | benralizumab | 10mg/30mg/100mg q8w | SC | COPD; had frequent exacerbations despite receiving guideline-based inhaled treatment; eosinophilic inflammation; | baseline blood eosinophil counts≥220 per cubic millimeter | ⑧ | 56 | 56 | 40-85 | 521/1024 |
| Ferguson2017^b^ | 61 | benralizumab | 30mg q4w | SC | mild to moderate, persistent asthma; weighing at least 40 kg; eosinophilic inflammation; | baseline blood eosinophil counts≥0.300 cells/mL | NR | 12 | 12 | 18-75 | 35/26 |
| FitzGerald2016^b^ | 728 | benralizumab | 30mg q4w/q8w | SC | uncontrolled, severe asthma; a history of two or more exacerbations in the previous year; weight 40 kg or heavier; eosinophilic inflammation; | baseline blood eosinophil counts≥0.300 cells/mL | ① or ⑩ | 56 | 60 | 12-75 | 442/286 |
| Haldar2009 | 61 | mepolizumab | 750mg q4w | IV | refractory eosinophilic asthma; a history of recurrent severe exacerbations; | a sputum eosinophil count of 3.0% or more | ⑦ | 48 | 50 | ≥18 | 29/32 |
| Harrison2021 | 656 | benralizumab | 30mg q8w | SC | severe eosinophilic asthma; at least 2 exacerbations in the previous year; eosinophilic inflammation; | baseline blood eosinophil counts≥0.150 cells/mL | ① or ④ | 24 | 24 | 18-75 | 399/257 |
| Jackson2022 | 290 | mepolizumab | 40mg/100mg q4w | IV | exacerbation-prone asthma; lived in a low-income area; at least two exacerbations treated with systemic corticosteroids in the previous year; required a minimum ICS; had been vaccinated for chickenpox; had documentation of current medical insurance; eosinophilic inflammation; | baseline blood eosinophil counts≥0.150 cells/mL | NR | 52 | 52 | 6-17 | 126/164 |
| Nair2017 | 220 | benralizumab | 30mg q4w/q8w | SC | asthma; treated with medium-dose to high-dose ICS and LABA therapy for at least 12 months; treated with high-dose ICS and LABA therapy for at least 6 months; eosinophilic inflammation; | baseline blood eosinophil counts≥0.150 cells/mL | ① | 24 | 36 | 18-75 | 135/85 |
| Noonan2013 | 210 | lebrikizumab | 125mg/250mg/500mg q4w | SC | asthma; not receiving ICS; eosinophilic inflammation; | ①IgE levels of greater than 100 IU/mL; ②baseline blood eosinophil counts≥ 0.14*10^9^ cells/L | NR | 12 | 20 | 18-65 | 134/76 |
| Ortega2014 | 576 | mepolizumab | 75mg/100mg q4w | IV/SC | asthma; at least two asthma exacerbations in the previous year; eosinophilic inflammation; | baseline blood eosinophil counts≥0.150 cells/mL at screening or ≥0.300 cells/ml at some time during the previous year | ① | 32 | 40 | 12-82 | 329/247 |
| Panettieri study1 2018^b^ | 199 | tralokinumab | 300mg q2w | SC | asthma; required medium to high dose ICS and a LABA for at least 3 months; eosinophilic inflammation; | FeNO ≥ 37 ppb | ① | 52 | 20 | 12-75 | 125/74 |
| Panettieri study2 2018^b^ | 229 | tralokinumab | 300mg q2w | SC | asthma; required medium to high dose ICS and a LABA for at least 3 months; eosinophilic inflammation; | FeNO ≥ 37 ppb | ① | 52 | 20 | 12-75 | 144/85 |
| Panettieri2020 | 233 | benralizumab | 30mg q4w | SC | severe asthma; treated with ICS/ LABA for ≥30 days; weighed ≥40 kg; eosinophilic inflammation; | baseline blood eosinophil counts≥0.300 cells/mL | NR | 12 | 16 | 18-75 | 157/76 |
| Park2016 | 103 | benralizumab | 2mg/20mg/100mg q8w | SC | eosinophilic asthma; treated with medium to high-dose ICS/LABA combination therapy for at least 1 year; had a documented history of 2–6 exacerbations requiring treatment with systemic corticosteroids in the past year; | FeNO ≥ 50 ppb | ① | 40 | 52 | 20-75 | 65/38 |
| Pavord study1 2017 | 462 | mepolizumab | 100mg q4w | SC | COPD; at least two moderate exacerbations or at least one severe exacerbation in the previous 12 months; eosinophilic inflammation; | baseline blood eosinophil counts≥0.150 cells/mL at screening or ≥0.300 cells/ml at some time during the previous year | ⑤ or ⑥ | 48 | 52 | ≥40 | 163/299 |
| Pavord study2 2017 | 674 | mepolizumab | 100/300mg q4w | SC | COPD; at least two moderate exacerbations or at least one severe exacerbation in the previous 12 months; eosinophilic inflammation; | baseline blood eosinophil counts≥0.150 cells/mL at screening or ≥0.300 cells/ml at some time during the previous year | ⑤ or ⑥ | 48 | 52 | ≥40 | 228/446 |
| Pavord2012 | 616 | mepolizumab | 75mg/250mg/750mg q4w | IV | asthma; at least two exacerbations  requiring systemic corticosteroid treatment in the previous year; eosinophilic inflammation | ① a sputum eosinophil count of 3.0% or more;②an exhaled nitric oxide concentration (FENO) of 50 ppb or more;③baseline blood eosinophil counts≥0.300 cells/mL | ① | 52 | 52 | 12-74 | 387/229 |
| Wenzel2013 | 104 | dupilumab | 300mg qw | SC | persistent, moderate-to-severe asthma; symptoms that were not well controlled with medium-dose to high-dose ICS plus LABA; eosinophilic inflammation; | ①baseline blood eosinophil counts≥0.300 cells/mL; ②a sputum eosinophil count of 3.0% or more | ① or ④ | 12 | 20 | 18-65 | 52/52 |
| Wenzel2016^b^ | 325 | dupilumab | 200mg q2w/300mg q4w | SC | asthma; treated with medium-to-high-dose ICS plus LABA; eosinophilic inflammation; | baseline blood eosinophil counts≥0.300 cells/mL | ① | 24 | 24 | ≥18 | 197/128 |
| Buhl2002 | 483 | omalizumab | 0.016 mg/kg IgE (IU/mL) q4w | SC | moderate-to-severe allergic asthma; | NR | ① | 28 | 28 | 12-76 | 244/239 |
| Busse2001 | 525 | omalizumab | 0.016 mg/kg IgE (IU/mL) q4w | SC | severe allergic asthma; | positive immediate responses on skin prick testing;  total serum IgE≥30 IU/mL to≤700 IU/mL | ① | 16 | 28 | 12-75 | 310/215 |
| Casale2015 | 365 | CYT003 | 0.3mg/1mg/2mg q2w | SC | persistent allergic asthma; | at least one positive aeroallergen-specific IgE (≥0.35 kU(A)/L), or a total serum IgE≥75 IU/mL | ① | 12 | 56 | 18-65 | 223/142 |
| Corren2017^b^ | 183 | tezepelumab | 70mg/210mg q4w/280mg q2w | SC | asthma; exacerbation history; eosinophilic inflammation; | baseline blood eosinophil counts≥0.40cells/mL | ① | 52 | 64 | 18-75 | NR |
| Hanania2011 | 848 | omalizumab | 0.016 mg/kg IgE (IU/mL) q4w | SC | severe allergic asthma; exacerbation history； | NR | ① | 48 | 48 | 12-75 | 557/291 |
| Harris2016 | 578 | quilizumab | 150mg/450mg q3m; 300mg qm | SC | atopic asthma; inadequately controlled; exacerbation history; | at least one positive aeroallergen-specific IgE (≥0.35 kU(A)/L), or a total serum IgE≥75 IU/mL | ① | 36 | 84 | 18-75 | 357/221 |
| Kulus2010 | 235 | omalizumab | 75 to 375 mg based on baseline serum total IgE qm | SC | moderate-to-severe allergic asthma; inadequately controlled; exacerbation history; | NR | ① | 52 | 52 | 6-12 | 81/154 |
| Lanier2003 | 460 | omalizumab | 0.016 mg/kg IgE (IU/mL) q4w | SC | severe allergic asthma; | NR | NR | 24 | 36 | 12-74 | 269/191 |
| Lanier2009 | 627 | omalizumab | 75 to 375 mg based on baseline serum total IgE qm | SC | moderate-to-severe allergic asthma; inadequately controlled; exacerbation history; | positive immediate responses on skin prick testing;  total serum IgE≥30 IU/mL to ≤1300 IU/mL | ① | 52 | 52 | 6-12 | 203/425 |
| Psallidas2021 | 81 | AZD1419 | 1mg/4mg/8mg qw | INH | moderate-to-severe asthma; ICS and LABA as the only maintenance treatment; eosinophilic inflammation; | baseline blood eosinophil counts≥0.25 cells/mL | NR | 12 | 52 | ≥18 | 46/35 |
| Rabe2021^b^ | 154 | itepekimab | 300mg q2w | SC | COPD; at least two exacerbations; | baseline blood eosinophil counts≥0.25 cells/mL | ⑧ | 52 | 72 | 40-75 | NR |
| Teach2015 | 348 | omalizumab | based on weight and serum IgE levels | SC | allergic asthma; exacerbation history; | positive immediate responses on skin prick testing;  total serum IgE levels suitable for omalizumab dosing; | ① | 12 | 12 | 6-17 | 115/233 |
| Yousuf2022^b^ | 81 | astegolimab | 490mg q4w | SC | COPD; at least two exacerbations; | baseline blood eosinophil counts≥0.300 cells/mL | ⑧ | 44 | 48 | ≥40 | NR |
| Menzies-Gow2021^b^ | 441 | tezepelumab | 210mg q4w | SC | asthma; exacerbation history; eosinophilic inflammation; | baseline blood eosinophil counts≥0.300 cells/mL | ① | 52 | 64 | 12-80 | NR |

Q2w every 2 weeks, IV intravenous infusion, SC subcutaneous injection, ICS inhaled corticosteroids, LABA long-acting β2-agonists, FEV1 forced expiratory volume in 1 s, NR not reported, ppb parts per billion, INH inhalation.

^a^ Exacerbation definition was at least one of the following criteria, ①worsening of asthma requiring emergency treatment, hospital admission, or three or more days of systemic corticosteroid treatment; ②a 20% or more decrease from baseline in FEV1; ③a two-times increase in the dose of either inhaled corticosteroids; ④a single injectable dose of corticosteroids; ⑤moderate, leading to systemic glucocorticoid treatment, antibiotic treatment, or both; ⑥severe, leading to hospitalization or resulting in death; ⑦treated with high-dose oral prednisolone for at least 5 days; ⑧a symptomatic worsening of COPD for at least 3 days resulting in any of the following outcomes: the use of systemic glucocorticoids, the use of antibiotics, or hospitalization or COPD-related death; ⑨a worsening of at least two major symptoms (dyspnea, sputum volume, and sputum purulence) or any major symptom together with one of several minor symptoms (sore throat, cold, fever without other cause, and increased cough or wheeze for more than 2 consecutive days); ⑩a temporary increase in a stable, background dosage of oral corticosteroids.

^b^ We only choose the data of eosinophilic

Supplementary Table 3: Univariable meta-regression analysis of study characteristics in exacerbation rate in asthma.

|  | Exacerbation rate in asthma | | | |
| --- | --- | --- | --- | --- |
|  | Coeffeicient | SE | 95%CI | P value |
| Limit of exacerbation history  (Yes *vs* No) | 0.96 | 0.14 | 0.71-1.29 | 0.773 |
| Criteria to assess 'Type 2 inflammation'  (Eosinophil counts *vs* IgE levels) | 1.21 | 0.17 | 0.90-1.63 | 0.185 |
| Age  (Adults *vs* including adolescents) | 1.01 | 0.16 | 0.73-1.41 | 0.948 |
| Sample size  (≤300 *vs* ＞300) | 1.01 | 0.16 | 0.72-1.41 | 0.953 |
| Risk bias  (High risk *vs* Unclear) | 1.10 | 0.18 | 0.77-1.55 | 0.590 |
| Anti-IgE agent  (Yes *vs* No) | 1.21 | 0.17 | 0.90-1.63 | 0.185 |
| Anti-TSLP agent  (Yes *vs* No) | 0.98 | 0.24 | 0.59-1.61 | 0.924 |
| TLR9 agonist  (Yes *vs* No) | 1.57 | 0.62 | 0.69-3.54 | 0.266 |
| Anti-IL-5 agent  (Yes *vs* No) | 0.81 | 0.11 | 0.62-1.07 | 0.140 |
| Anti-IL-4/13 agent  (Yes *vs* No) | 0.91 | 0.25 | 0.52-1.59 | 0.733 |
| Severity  (Severe *vs* Modoreate-to-severe) | 0.82 | 0.11 | 0.62-1.09 | 0.166 |
| Atopic  (Yes *vs* No) | 1.19 | 0.18 | 0.87-1.62 | 0.252 |

SE standard error, CI confidence interval

Supplementary Table 4: GRADE evidence profile

| Quality assessment | | | | | | No. of patients | RR/SMD  (95%CI) | P value | Quality |
| --- | --- | --- | --- | --- | --- | --- | --- | --- | --- |
| No. of studies | Risk of bias | Inconsistency | Indirectness | Imprecision | Other considerations |  |  |  |  |
| **Exacerbation rate in COPD** | | | | | | | | | |
| 7 | no serious risk of bias | serious inconsistency ^a^ | no serious indirectness | no serious imprecision | none | 4,137 | 0.89  (0.83-0.95) | P ＜0.001 | ⊕⊕⊕O  MODERATE |
| **Exacerbation rate in asthma** | | | | | | | | | |
| 25 | no serious risk of bias | serious inconsistency ^a^ | no serious indirectness | no serious imprecision | reporting bias ^b^ | 10,466 | 0.59  (0.52-0.68) | P ＜0.001 | ⊕⊕OO  LOW |
| **FEV1 in COPD** | | | | | | | | | |
| 4 | no serious risk of bias | serious inconsistency ^a^ | no serious indirectness | no serious imprecision | reporting bias ^b^ | 2,920 | 0.05  (-0.01-0.10) | P = 0.086 | ⊕⊕OO  LOW |
| **FEV1 in asthma** | | | | | | | | | |
| 22 | no serious risk of bias | serious inconsistency ^a^ | no serious indirectness | no serious imprecision | none | 8,305 | 0.09  (0.08-0.11) | P ＜0.001 | ⊕⊕⊕O  MODERATE |

^a^ There is controversy in different studies. ^b^ Egger's test P＜0.05. RR rate ratio, SMD standard mean difference, CI confidence interval.

Supplementary Table 5: PRISMA 2020 checklist

| **Section and Topic** | **Item #** | **Checklist item** | **Location where item is reported** |
| --- | --- | --- | --- |
| **TITLE** | | |  |
| Title | 1 | Identify the report as a systematic review. | 1 |
| **ABSTRACT** | | |  |
| Abstract | 2 | See the PRISMA 2020 for Abstracts checklist. | 1 |
| **INTRODUCTION** | | |  |
| Rationale | 3 | Describe the rationale for the review in the context of existing knowledge. | 2 |
| Objectives | 4 | Provide an explicit statement of the objective(s) or question(s) the review addresses. | 2 |
| **METHODS** | | |  |
| Eligibility criteria | 5 | Specify the inclusion and exclusion criteria for the review and how studies were grouped for the syntheses. | 3 |
| Information sources | 6 | Specify all databases, registers, websites, organisations, reference lists and other sources searched or consulted to identify studies. Specify the date when each source was last searched or consulted. | 3 |
| Search strategy | 7 | Present the full search strategies for all databases, registers and websites, including any filters and limits used. | 3 |
| Selection process | 8 | Specify the methods used to decide whether a study met the inclusion criteria of the review, including how many reviewers screened each record and each report retrieved, whether they worked independently, and if applicable, details of automation tools used in the process. | S1 |
| Data collection process | 9 | Specify the methods used to collect data from reports, including how many reviewers collected data from each report, whether they worked independently, any processes for obtaining or confirming data from study investigators, and if applicable, details of automation tools used in the process. | 3 |
| Data items | 10a | List and define all outcomes for which data were sought. Specify whether all results that were compatible with each outcome domain in each study were sought (e.g. for all measures, time points, analyses), and if not, the methods used to decide which results to collect. | 3 |
|  | 10b | List and define all other variables for which data were sought (e.g. participant and intervention characteristics, funding sources). Describe any assumptions made about any missing or unclear information. | 3 |
| Study risk of bias assessment | 11 | Specify the methods used to assess risk of bias in the included studies, including details of the tool(s) used, how many reviewers assessed each study and whether they worked independently, and if applicable, details of automation tools used in the process. | 3 |
| Effect measures | 12 | Specify for each outcome the effect measure(s) (e.g. risk ratio, mean difference) used in the synthesis or presentation of results. | 3 |
| Synthesis methods | 13a | Describe the processes used to decide which studies were eligible for each synthesis (e.g. tabulating the study intervention characteristics and comparing against the planned groups for each synthesis (item #5)). | 3 |
|  | 13b | Describe any methods required to prepare the data for presentation or synthesis, such as handling of missing summary statistics, or data conversions. | 4 |
|  | 13c | Describe any methods used to tabulate or visually display results of individual studies and syntheses. | 4 |
|  | 13d | Describe any methods used to synthesize results and provide a rationale for the choice(s). If meta-analysis was performed, describe the model(s), method(s) to identify the presence and extent of statistical heterogeneity, and software package(s) used. | 4 |
|  | 13e | Describe any methods used to explore possible causes of heterogeneity among study results (e.g. subgroup analysis, meta-regression). | 4 |
|  | 13f | Describe any sensitivity analyses conducted to assess robustness of the synthesized results. | 6 |
| Reporting bias assessment | 14 | Describe any methods used to assess risk of bias due to missing results in a synthesis (arising from reporting biases). | 6 |
| Certainty assessment | 15 | Describe any methods used to assess certainty (or confidence) in the body of evidence for an outcome. | 7 |
| **RESULTS** | | |  |
| Study selection | 16a | Describe the results of the search and selection process, from the number of records identified in the search to the number of studies included in the review, ideally using a flow diagram. | 4 |
|  | 16b | Cite studies that might appear to meet the inclusion criteria, but which were excluded, and explain why they were excluded. | 4 |
| Study characteristics | 17 | Cite each included study and present its characteristics. | 4 |
| Risk of bias in studies | 18 | Present assessments of risk of bias for each included study. | 6 |
| Results of individual studies | 19 | For all outcomes, present, for each study: (a) summary statistics for each group (where appropriate) and (b) an effect estimate and its precision (e.g. confidence/credible interval), ideally using structured tables or plots. | 5 |
| Results of syntheses | 20a | For each synthesis, briefly summarise the characteristics and risk of bias among contributing studies. | 5 |
|  | 20b | Present results of all statistical syntheses conducted. If meta-analysis was done, present for each the summary estimate and its precision (e.g. confidence/credible interval) and measures of statistical heterogeneity. If comparing groups, describe the direction of the effect. | 5 |
|  | 20c | Present results of all investigations of possible causes of heterogeneity among study results. | 5 |
|  | 20d | Present results of all sensitivity analyses conducted to assess the robustness of the synthesized results. | 6 |
| Reporting biases | 21 | Present assessments of risk of bias due to missing results (arising from reporting biases) for each synthesis assessed. | 6 |
| Certainty of evidence | 22 | Present assessments of certainty (or confidence) in the body of evidence for each outcome assessed. | 6 |
| **DISCUSSION** | | |  |
| Discussion | 23a | Provide a general interpretation of the results in the context of other evidence. | 7 |
|  | 23b | Discuss any limitations of the evidence included in the review. | 7 |
|  | 23c | Discuss any limitations of the review processes used. | 7 |
|  | 23d | Discuss implications of the results for practice, policy, and future research. | 8 |
| **OTHER INFORMATION** | | |  |
| Registration and protocol | 24a | Provide registration information for the review, including register name and registration number, or state that the review was not registered. | 2 |
|  | 24b | Indicate where the review protocol can be accessed, or state that a protocol was not prepared. | 2 |
|  | 24c | Describe and explain any amendments to information provided at registration or in the protocol. | 2 |
| Support | 25 | Describe sources of financial or non-financial support for the review, and the role of the funders or sponsors in the review. | 8 |
| Competing interests | 26 | Declare any competing interests of review authors. | 8 |
| Availability of data, code and other materials | 27 | Report which of the following are publicly available and where they can be found: template data collection forms; data extracted from included studies; data used for all analyses; analytic code; any other materials used in the review. | 8 |

Supplementary Table 6: PRISMA 2020 for abstracts checklist

| **Section and Topic** | **Item #** | **Checklist item** | **Reported (Yes/No)** |
| --- | --- | --- | --- |
| **TITLE** | | |  |
| Title | 1 | Identify the report as a systematic review. | YES |
| **BACKGROUND** | | |  |
| Objectives | 2 | Provide an explicit statement of the main objective(s) or question(s) the review addresses. | YES |
| **METHODS** | | |  |
| Eligibility criteria | 3 | Specify the inclusion and exclusion criteria for the review. | YES |
| Information sources | 4 | Specify the information sources (e.g. databases, registers) used to identify studies and the date when each was last searched. | YES |
| Risk of bias | 5 | Specify the methods used to assess risk of bias in the included studies. | YES |
| Synthesis of results | 6 | Specify the methods used to present and synthesise results. | YES |
| **RESULTS** | | |  |
| Included studies | 7 | Give the total number of included studies and participants and summarise relevant characteristics of studies. | YES |
| Synthesis of results | 8 | Present results for main outcomes, preferably indicating the number of included studies and participants for each. If meta-analysis was done, report the summary estimate and confidence/credible interval. If comparing groups, indicate the direction of the effect (i.e. which group is favoured). | YES |
| **DISCUSSION** | | |  |
| Limitations of evidence | 9 | Provide a brief summary of the limitations of the evidence included in the review (e.g. study risk of bias, inconsistency and imprecision). | YES |
| Interpretation | 10 | Provide a general interpretation of the results and important implications. | YES |
| **OTHER** | | |  |
| Funding | 11 | Specify the primary source of funding for the review. | YES |
| Registration | 12 | Provide the register name and registration number. | YES |
